# Testing the Role of Emotion Dysregulation as a Predictor of Juvenile Recidivism

**DOI:** 10.3390/ejihpe11010007

**Published:** 2021-01-21

**Authors:** Kalin Z. Salinas, Amanda Venta

**Affiliations:** 1 Department of Philosophy & Psychology, Sam Houston State University, Huntsville, TX 77340, USA; 2 Department of Psychology, University of Houston, Houston, TX 77004, USA; aventa@uh.edu

**Keywords:** emotion regulation, recidivism, delinquency, adolescents, crime

## Abstract

The current study proposed to determine whether adolescent emotion regulation is predictive of the amount and type of crime committed by adolescent juvenile offenders. Despite evidence in the literature linking emotion regulation to behaviour problems and aggression across the lifespan, there is no prior longitudinal research examining the predictive role of emotion regulation on adolescent recidivism, nor data regarding how emotion regulation relates to the occurrence of specific types of crimes. Our primary hypothesis was that poor emotion regulation would positively and significantly predict re-offending among adolescents. We tested our hypothesis within a binary logistic framework utilizing the Pathways to Desistance longitudinal data. Exploratory bivariate analyses were conducted regarding emotion regulation and type of crime in the service of future hypothesis generation. Though the findings did not indicate a statistically significant relation between emotion regulation and reoffending, exploratory findings suggest that some types of crime may be more linked to emotion regulation than others. In sum, the present study aimed to examine a hypothesized relation between emotion regulation and juvenile delinquency by identifying how the individual factor of dysregulated emotion regulation may have played a role. This study’s findings did not provide evidence that emotion regulation was a significant predictor of recidivism over time but did suggest that emotion regulation is related to participation in certain types of crime one year later. Directions for future research that build upon the current study were described. Indeed, identifying emotion regulation as a predictor of adolescent crime has the potential to enhance current crime prevention efforts and clinical treatments for juvenile offenders; this is based on the large amount of treatment literature, which documents that emotion regulation is malleable through treatment and prevention programming.

## 1. Introduction

Adolescents are committing crime at an alarming rate despite decreases in arrest rates [1,2]. The broader literature regarding adolescent delinquent behaviour has identified many individual factors that contribute to problem behaviours in adolescents, including emotion dysregulation [3]. While this factor has been shown to contribute to behavioural problems in adolescents, little research has examined dysregulated emotion regulation (DER) in the context of recidivism and/or specific types of crime committed by adolescents. The present study proposed to test DER as a predictor of reoffending and type of crime committed by juvenile offenders using archival data from a longitudinal study. Examining this predictor has the potential to enhance current crime prevention efforts and clinical treatments for juvenile offenders by identifying how the individual factor of emotion dysregulation may play a role.

The current study was guided by Gratz and Romer’s [4] model of emotion dysregulation, which has been shown to be valid for use in adolescents [5,6]. This model defines DER as a process that includes: (a) lack of awareness, understanding, and acceptance of emotions; (b) lack of clarity of emotional responses; (c) nonacceptance of emotional responses; (d) limited access to emotion regulation (ER) strategies perceived as effective; (e) difficulties controlling impulses when experiencing negative emotions; and (f) difficulties engaging in goal-directed behaviour when experiencing negative emotions. Impairments in ER have been linked to adolescents’ academic performance [7] and mental health [8], broadly, with ER often acting as a mediator. For instance, early onset persistent delinquent youth were shown to fall below average on general intellectual abilities, with ER strongly mediating the relation between verbal learning and psychopathology. Similarly, a study conducted by King et al. [8] found that witnessing community violence and/or home violence predicted poorer achievement over time among African American adolescents, but that adolescents experienced a decline in school grades only when poor ER skills were identified. King et al. [8] concluded that ER skills were protective against a decrease in academic performance for adolescents who witnessed community violence. In both studies, the significant mediational role of ER indicates that it could be an effective intervention target for improved academic success and social-emotional learning in adolescents. The current study sought to uncover the role of ER in the context of adolescent recidivism, with the hope of identifying a malleable treatment target (i.e., ER) that can be used to reduce adolescent recidivism.

## 2. Literature Review

### 2.1. Delinquency and Emotion Dysregulation

Impaired ER has been repeatedly linked to childhood behaviour problems. For instance, Schoorl et al. [9] examined ER difficulties in boys who suffered from oppositional defiant disorder (ODD) and/or conduct disorder (CD). Researchers administered an ultimatum game to a group of boys aged eight to twelve; results from this game showed that boys with ODD and CD rejected more ambiguous offers than non-diagnosed boys, which was conceptualized by the authors as an indication of worsened ER. The same study found that parents of boys with ODD and CD reported that their children experienced more ER difficulties in daily life, although the boys themselves did not recognize the ER deficits. Likewise, Cavanagh et al. [10] found that ODD and CD were strongly and significantly correlated with DER. It was advised that children who exhibit disruptive behaviours should be examined for dysregulation of emotions [6]. Indeed, the inability to regulate emotions is a distinguishing feature of CD [11]. In particular, children with CD show ER deficits specific to their ability to regulate anger [11,12,13,14].

In some cases, early onset behavioural problems may linger and lead to serious consequences like juvenile delinquency, which are related to ER [3,15]. Kemp et al. [3] examined the association between ER and future arrests in adolescents from urban public schools; researchers found a strong relation between ER and future arrests. One explanation for this relation was that youth with impaired ER skills may partake in illegal acts due to poor judgment and risky decision-making driven by DER. Kemp et al. [3] also suggested that the origin of arrests may be due to negative interactions with law enforcement that result from DER. Adolescents who can effectively manage their emotional responses may be less likely to have law enforcement interactions end with arrests. On the other hand, adolescents who have poor emotional management may be perceived by law enforcement as more at risk to commit criminal offenses, which could evoke arrests [3]. Additionally, DER has been linked to forms of delinquency other than arrests. Pihet et al. [16], for instance, illustrated that minor rule breaking was associated with deficits in ER and impulsivity. Furthermore, Rawana et al. [17] illustrated that substance abuse and impulsivity in adolescents with mental health concerns were associated with maladaptive ER.

### 2.2. ER and Aggression

Bolstering the data linking ER and delinquency is a broader section of the literature, documenting associations between ER and aggression. Some of this research has focused on the ability to manage anger, specifically. For instance, Cooley et al. [18] illustrated that the inability to regulate anger influences relations between peer victimization and aggression among children between the ages of eight and twelve. Further investigations illustrated that high levels of anger regulation—reliant on broader ER—reduced the strength of the relation between child-reported peer victimization and physical aggression. Another finding illustrated by Cooley et al. [18] indicated that high levels of anger regulation were associated with a weaker relation between child-reported peer victimization and relational aggression. Finally, Shorey et al.’s [19] study on undergraduate women found that both trait anger and ER difficulties were positively associated with psychological aggression with women who resorted to psychological aggression reporting more trait anger and ER difficulties than women who did not. Another finding suggested that difficulties in ER were associated with higher trait anger, which predicted psychological aggression [19]. This led to the conclusion that deficits in ER may lead individuals to experience more anger; this anger may arise in response to frustrations about not being able to be in control of their emotions, leading to aggressive behaviours towards others [19].

Other studies have shown that ER difficulties in general (i.e., not anger regulation specifically) act as a contributor to aggression [20] and aggressive behaviours [21]. Indeed, individuals with DER exhibit more aggressive behaviours when compared to individuals without DER [22]. For instance, Garofalo et al. [20] found that negative emotionality and all six dimensions of ER (i.e., awareness, clarity, nonacceptance, impulse, goals, and strategies) were associated with physical aggression [4]. ER explained variance in physical aggression more so than negative emotionality [4]. Similarly, both negative emotionality and DER played a role in contributing to the aggressive habits of violent offenders [20]. ER was also associated with aggression in veterans [23]. Miles et al. [23] illustrated that under-regulation and over-regulation of negative emotions was associated with aggression in veterans with post-traumatic stress disorder (PTSD). Similarly, Roberton et al. [22] illustrated that DER was able to explain incremental variance in aggressive behaviours over and above anger. Roberton et al. [24] found that individuals with maladaptive ER exhibited increased levels of aggression and more prominent histories of aggression, as opposed to individuals who had adaptive ER. A more recent study by Alleyne and Parfitt [21] found that animal abuse was related to DER in adults; individuals exhibited decreases in impulse control and more instances of aggressive behaviours towards animals when DER was present. In youth, Grisso [25] illustrated that children with many mental disorders (including CD) are more likely to display aggression when their emotions are dysregulated.

### 2.3. ER Treatment in Justice-Involved Groups

Data documenting the positive effects of ER treatments on aggression and delinquency further support a link between these constructs, as there have been successful interventions for adults who suffer from DER and who have already committed acts of violent crime. Garofalo et al.’s [20] study indicated that ER skills may buffer the positive relation between negative emotionality and aggression in violent offenders in prison. These findings suggest a positive outlook for the treatment of offenders who suffer from DER. Furthermore, interventions for aggression should include aspects of emotion regulation [26], specifically, for violent offenders who experience frequent negative emotionality from their experiences [6].

Keiley et al. [27] conducted a study to investigate whether Multiple-Family Group Intervention (MFGI) was effective in reducing problem behaviours (i.e., externalizing) in a population of incarcerated youth. Results showed that MFGI did help to decrease problem behaviours, including maladaptive ER. The decreases in problem behaviours were primarily due to significant decreases in maladaptive ER, which continued up to a year after the intervention. Although the current study does not examine the effects of intervention on this population of adolescents, treatment modalities for individuals with DER may be vital to examine in future studies if our hypotheses are correct.

### 2.4. Areas of Inconsistency in Prior Research

Despite the aforementioned literature linking ER deficits to delinquency and related outcomes, it is notable that parallel literatures documents superior ER in some delinquent groups. For instance, a study done by Forslund et al. [28] found that negative emotionality showed a specific link to conduct problems in a group of developing children. This study used parental ratings of ER, emotionality, and externalizing behaviours and investigated how these factors related to conduct problems. Negative emotionality showed a link to conduct problems, while regulation of negative emotions did not [28]. Similarly, children with high callous/unemotional traits (CU) were assessed as a specific subgroup of children with conduct problems [29]. This study had young children, divided into high vs. low CU traits, watch movie sequences of fearful, attachment related, and neutral stimuli and their ER strategies were examined. Results showed that high CU children were able to disengage from the fear stimuli by showing more happiness to a brief “slapstick” interlude in the movie [29]. The same group of children expressed similar trends towards higher emotional responses and ER strategies in an attachment scenario from the same movie. High CU children used disengagement more than others and were equally likely than the non-CU children to use disengagement to cope with the emotional nature of the attachment scene. The current study aims to elucidate this relation between ER and delinquency in a population of adolescent offenders, to invalidate the findings of Forslund et al. [28] and Dadds et al. [29].

### 2.5. Limitation in Prior Research

Many of the aforementioned studies identified significant links between ER and behavioural problems, aggression, or conduct problems, though this literature is not without significant limitations. First, only one study, to our knowledge, examined this association prospectively, linking ER and future arrests [3]. This study focused on adolescents from urban public schools, rather than justice-involved adolescents. Second, a majority of the studies linking ER and crime rates did so using adult samples, limiting the utility of this literature base in the context of adolescent offending. Third, the aforementioned studies did not identify the type of crime being predicted by ER. Type of crime associated with ER is important to identify in order to target specific populations of juveniles and administer appropriate interventions to reduce the crime that is most associated with DER. Fourth, a majority of the studies reviewed had participants whose demographics limit generalizability and sample sizes that were too small to generalize to a greater population; such as Miller et al.’s [30] study which had a small sample size (*n* = 94) and participants in the study were primarily Caucasian. Other studies utilized participants from specific racial backgrounds and/or age ranges; a majority of studies used adult participants only. For example, Garofalo et al. [20] used violent offenders who were in Italian prisons in their study. Koch et al. [31] only used participants from German prisons who were primarily German (95.1%). Although these research studies contributed important information to the ER literature, their limitations hinder the possibility of enhancing pre-existing treatments for a greater population of adolescents who may suffer from ER deficits.

### 2.6. Current Study

In order to address these limitations, research is needed examining ER and juvenile crime in a large, diverse sample. To summarize, there is little (or no) research directly examining the relation between ER and adolescent recidivism and/or types of crimes committed, and, further, little existing research with any age group is longitudinal. Broadly, this study proposed to determine whether adolescent ER was predictive of reoffending and types of crime committed with the use of the Pathways to Desistance [32] longitudinal data. Our primary hypothesis was that emotion dysregulation would positively and significantly predict re-offending among adolescents. Based on aforementioned literature linking DER to both aggression and impulsivity, it was expected that DER would be a significant, positive predictor of all types of crime; the relative strength of these relations was explored for future hypothesis generation.

## 3. Method

### 3.1. Participants

This study used publicly available data from the Pathways to Desistence study [32]. The Pathways to Desistance is a longitudinal and collaborative project that followed 1354 serious juvenile offenders for 7 years after their initial conviction [33]. Participants were recruited from Maricopa County, Arizona or Philadelphia, Pennsylvania. To be eligible for the study participants had to be between the ages of 14 and 18 at the time they committed the offense, they had to have been found guilty of a serious offense, and they had to provide informed assent and have parental consent. For this study, a serious offense included felonies as well as some misdemeanours (i.e., property offenses, sexual assault, or weapons offenses). Descriptive statistics of participant characteristics can be found in Table 1. There was a cap at 15% for male offenders who were found guilty of a drug charge to avoid an over-representation of drug offenders. Females who met the previous criteria (i.e., age and crime) were considered candidates for enrolment as well as adolescents being considered for trial in an adult judicial system. There was a 20% decline rate for participation.

### 3.2. Measures

*Children’s Emotional Intensity Child Report* [34]. This is a self-reported measure of the participants’ ability to regulate their emotions. This scale contains nine items answered on a 6-point Likert scale ranging from 0 (disagree) to 5 (agree) (or true/false, or yes/no, etc.). A sample item from this scale includes: “I know things to do to make myself more happy.” In the Pathways to Desistance study, internal consistency for this scale was high (baseline alpha = 0.81). This instrument has also been utilized in previous research with adolescents [35], demonstrating adequate psychometric properties. In the Pathways study, this assessment was completed by the juvenile and the collateral informant, but only juvenile data is publicly available. For the current study, the baseline score from youth reporters was utilized as an independent variable.

*Self-reported offending* [36,37]. This measure was developed for the Pathways study to assess the participant’s account of their involvement in criminal and antisocial activities. The scale has a total of 24 items that provide information about the types of crime the participant has engaged in since the last assessment. For each item that was endorsed, a set of follow-up questions were administered to collect more information about the particular offense. These follow-up questions provide additional information including time of crime, age of onset, and if the act was committed alone or with others. For this study, offending (yes/no) and type of crime assessed at 6-, 12-, 18- and 24-month follow-ups were utilized as dependent variables.

### 3.3. Procedures

Archival data from the Pathways study relied on self-report from the youth who participated. All of the youth in the facilities completed a series of interviews, including baseline interviews (in November 2000; and the last baseline interview was completed in March 2003) and follow-up interviews. The baseline interview obtained information about each youth’s background characteristics, aspects of individual functioning, psychosocial development, information about their family, personal relationships, and about their community. Considering the length of the interview, researchers broke the interview into two, 2-hour sessions; lessening the possibility of fatigue. The collateral interviews were completed at the same time as the baseline interviews and then annually for the first three years of the follow-up interviews, though collateral data is not available for public download. After the youth completed the baseline interviews, they began the follow-up interviews; all youth participated in follow-up interviews at 6, 12, 18, 24, 30, 36, 48, 60, 72, and 84 months (i.e., 2000 to 2007). Youth completed follow-up interviews every six months during the first three years of the study and then annually for the following 84 months. Furthermore, each interview date was calculated based on the date of the first interview (baseline). These interviews were shorter than the baseline interviews (2 h) and participants were paid with a “graduated payment system” ranging from $50 to $115 (depending on the length of the interview). If follow-up interviews were not completed between 6 weeks before or 8 weeks after the targeted date, then it was considered a “missed” data point (interview).

The data from this study was collected with the use of computers and they took place either in the participants’ homes, libraries, or in their correctional facilities. The measures and patterns associated with their measures were all programmed onto laptop computers. During the interview, trained individuals read every item out loud and participants then had the choice to respond by keypad or verbally. Confidentiality was established through the Department of Justice. Self-report information was validated through interviews with collaterals and information from official records (i.e., FBI records of the arrests and court records from each jurisdiction). Repeated assessments were made of participants’ psychological development, behaviour, social relationships, mental health, and their experiences in the justice system. The interviews were conducted over seven years after being convicted of a criminal offense.

For the current study, approval from the SHSU Institutional Review Board was attained. Archival data was then downloaded, and the described variables were saved into a dataset for analysis.

### 3.4. Data Analytic Plan

The current study proposed one main hypothesis: that DER would positively and significantly predict re-offending among adolescents. Prior to empirically examining this question, potential confounding variables (e.g., age and gender) were examined and assumptions of normality were examined. Those variables that demonstrated a significant relation with the outcome variable were included as covariates. In order to test the main study hypothesis, a repeated measures general linear model was planned. However, based on problematic non-normality in the dependent variable, self-reported offending was dichotomized, and binary logistic regression was used in four separate models (i.e., 6-, 12-, 18-, and 24-month offending). The main study hypothesis was tested by examining DER as a predictor in each model, this term was expected to be positive and significant indicating a link between DER and recidivism.

Based on aforementioned literature linking DER to both aggression and impulsivity, it was expected that DER would be a significant, positive predictor of all types of crime; the relative strength of these relations was explored for future hypothesis generation utilizing analyses of variance (i.e., type of crime based on mean ER).

## 4. Results

### 4.1. Confirmatory Analyses

Descriptive statistics (i.e., mean, SD, range) for all continuous variables (i.e., age at first offense, ER, SRO continuous) and dichotomous variables (i.e., gender and SRO dichotomous) are reported in Table 1. Notably, descriptive statistics indicated problematic positive skewness and leptokurtic kurtosis for all self-reported offending (SRO) continuous variables. To assess the main study hypothesis, a repeated measures general linear model was planned and pre-registered in accordance with the open science initiative. However, we were surprised to find that the SRO variables could not be used in dimensional analyses (due to lack of normality and poor fit with Poisson and negative binomial models). Thus, binary logistic regression was used in four separate models that were not pre-registered. Regarding dichotomized SRO, the following percentage of participants endorsed reoffending at each timepoint: 54.2% at 6 months, 47.6% at 12 months, 42.8% at 18 months, and 40.5% at 24 months. Independent samples *t*-tests indicated that SRO was not related to age at first offense: 6 months (*p* = 0.798), 12 months (*p* = 0.781), 18 months (*p* = 0.679), and 24 months (*p* = 0.717). Chi-square analyses indicated that SRO was not related to gender: 6 months (*p* = 0.095), 18 months (*p* = 0.453), and 24 months (*p* = 0.356). Chi-square analyses indicated that SRO was related to gender at 12 months (*p* = 0.026). Independent samples *t*-tests indicated that SRO was not related to ER at baseline: 6 months (*p* = 0.896), 12-months (*p* = 0.734), 18-months (*p* = 0.123), and 24-months (*p* = 0.307).

### 4.2. Exploratory Analyses: Regression Analyses

Four binary logistic regressions were used to test the relation of ER and identified covariates on SRO across time in separate analyses. In each regression, the following variables were added as independent variables: ER at baseline and gender. Based on this assessment, all continuous independent variables were found to be linearly related to the logit of the dependent variable. Dichotomized SRO was entered as a dependent variable in separate regressions (i.e., 6, 12, 18, and 24 months).

Regression findings are fully depicted in Table 2. In the first regression, with 6-month SRO as the dependent variable, the overall model was not significant (*X*^2^ (2) = 1.937, *p* = 0.380). Of the two predictor variables, neither was statistically significantly related to SRO (as shown in Table 2). In the second regression, with 12-month SRO as dependent variable, the overall model was not significant (*X*^2^ (2) = 4.179, *p* = 0.124). Here, only gender was a significant predictor of SRO (*β* = 0.713, *SE* = 0.169, *p* = 0.045) such that men were more likely to re-offend than women. In the third regression, with 18-month SRO as dependent variable, the overall model was not significant (*X*^2^ (2) = 2.446, *p* = 0.294). Predictor variables were not statistically significantly related to SRO. In the fourth regression, with 24-month SRO as dependent variable, the overall model was not significant (*X*^2^ (2) = 1.215, *p* = 0.545). No predictor variables were statistically significantly related to SRO. An additional analysis was conducted to determine if unique offenders had ER scores that were predictive of SRO at each timepoint. Unique offenders were defined as juveniles that had not re-offended at any previous timepoints, such that offenders at the 12-month follow up who had offended at the 6-month follow up were removed from the 12-month SRO timepoint. We identified the unique offenders and ran a binary logistic regression with the remaining offenders. The overall models for each timepoints were not statistically significant. A second additional analysis was conducted testing the role of ER as a predictor of aggressive crime types only; however, this analysis did not result in statistically significant findings.

### 4.3. Exploratory Analyses: ER & Type of Crime

Independent-samples *t*-tests were conducted to determine if there were differences in ER between individuals who committed different types of crime throughout the four time points (i.e., 6, 12, 18, and 24 months). A total of 22 types of crime were utilized, as more serious crimes (i.e., murder, rape, etc.) were not available due to their sensitive nature and protection of confidentiality. *T*-test findings are depicted in Table 3. Statistically significant differences in ER were exhibited at the 12-month time point on the following crime types: using a check or credit card illegally, *t*(1258) = −2.01, *p* = 0.045, 95% CI [−0.64, −0.01]; carjacking, *t*(1258) = −2.16, *p* = 0.031, 95% CI [−0.79, −0.04]; paid for sexual relations, *t*(1258) = −2.27, *p* = 0.023, 95% CI [−1.14, −0.08]; and shot at someone (bullet hit), *t*(1258) = −1.99, *p* = 0.046, 95% CI [−0.49, 0.11], although mean differences were very small.

## 5. Discussion

The goal of the present study was to determine whether adolescent ER could predict reoffending and type of crimes committed by juvenile offenders. Our primary hypothesis was that DER would positively and significantly predict re-offending among adolescents. The results of the current study reveal that ER was not a statistically significant predictor of juvenile recidivism across time in this population of adolescent criminal offenders, in contrast to our primary hypothesis. In this sample, participants who recidivated did not show differences in baseline ER from those who did not at any of the four timepoints (6-, 12, 18, and 24 months). It should be noted, however, that planned, pre-registered confirmatory hypotheses and analyses could not be tested due to non-normality in the dependent variable (i.e., self-reported offending) and, thus, analyses were exploratory in nature and utilized a dichotomized outcome variable.

The current findings are not in line with the findings of previous research. For example, Kemp et al. [3] examined the relation between ER and future arrests in a group of adolescents from urban public schools. Indeed, they found a strong relation between ER and future arrests and suggested that youth with impaired ER may participate in illegal acts due to their poor judgment and risky decision making—driven by these dysregulated emotions. Here, differences in findings may be due to the stages of delinquency and the measures utilized in each study. Specifically, Kemp et al. [3] utilized the Difficulties in Emotion Regulation Scale (DERS) to identify emotion regulation skills—highlighting the Lack of Emotional Awareness and the Limited Access to Emotion Regulation Strategies subscales. The DERS was shown to have strong reliability in their study [3] and has been validated in adolescent samples (34, 47, 51). The present study utilized the Pathways to Desistance longitudinal dataset, which used the Children’s Emotion Regulation scale (CER). CER scale was shortened to 12 items from its original format of 33 items for it to be utilized as a self-report measure in the Pathways study. Further analysis of CER scale by the Pathways study authors indicated psychometric problems requiring refinement, resulting in additional decreases in the number of items presented—the final measure contained only 9 of the 33 items from the CER scale. This 9-item version yielded adequate fit to the baseline data (*alpha* = 0.81; *NFI* = 0.951, *NNFI* = 0.938, *CFI* = 0.958, *RMSEA* = 0.06; [32]). To our knowledge, the validity and reliability of this 9-item CER scale has not been tested as a measure of ER in adolescents outside of the Pathways study. Poor coverage of the ER construct in the 9-item CER may explain the discrepancy in findings between the current study and prior research. Further, Kemp et al. [3] utilized a sample of seventh graders from public schools who were suspected of having behavioral or emotional deficits but had no previous arrest records. Our study utilized a sample of adolescents who have already committed at least one serious offense—quite a large difference in sample characteristics and one that may have led to restriction of range in ER. Indeed, the narrow range of the CER, in combination with the generally severe nature of disturbance in the current sample may have obscured relations between ER and offending that exist in the broader population.

Another prior study, conducted by Pihet et al. [16], illustrated that minor rule breaking was associated with emotion dysregulation and impulsivity. Like Kemp et al. [3], this study did not examine known juvenile offenders, possibly explaining why they were able to detect a relation between rule breaking and emotion dysregulation. Further, Pihet et al. [16] did not utilize measures specific to ER as whole, instead, they used the Toronto Alexithymia Scale; measuring the difficulty to identify feelings and externally oriented thinking [16]. Pihet et al.’s [16] measure of “alexithymia” may be measuring a similar subtype of ER as Kemp et al.’s [13] measure of “lack of emotional awareness.” Indeed, the Toronto Alexithymia Scale measures a participant’s inability to understand and describe emotions/feelings. Similarly, items on the lack of awareness subscale for the DERS measure the understanding, processing, and identifying of emotions. The present study’s measure, the CER, seems to be identifying adolescents’ ability to change intense emotions—items include “I can change my feelings by thinking of something else” [32]. It seems that the present study is utilizing a qualitatively different subtype of ER than that assessed in the two aforementioned studies. It may be that this facet of ER utilized in both studies [3,16] is relevant to the prediction of future criminal behaviour and recidivism, even in juvenile offenders, but was not assessed in the Pathways to Desistance study.

Still, some links between ER and recidivism were observed in the current study utilizing exploratory analyses. Indeed, regarding the specific types of crime committed by juvenile offenders in relation to ER scores, there were few instances where baseline ER was statistically different between juvenile reoffenders and non-reoffenders. All links between ER and recidivism were observed at the 12-month timepoint, suggesting that baseline ER may play a predictive role in recidivism in some specific domains of crime. The criminal offenses with statistically significant differences in ER group means between juveniles who recidivated and those who did not include used check or credit card illegally; carjacking; paying for sexual relations and having shot at someone where the bullet injured the individual. All four of these criminal offenses are listed under the “antisocial acts” category on the pathways to desistance website [32]. Using a check or credit card illegally and paying for sexual relations both fell under the income offending category; whereas having shot at someone where the bullet hit the individual fell under the aggressive offense category [32]. Carjacking did not fall under any category related to the other three types of crimes. A study done by Walter [38] found that social deviance and disinhibition predicted income offending in youth who scored low on core interpersonal-affective traits of psychopathy using the Pathways to Desistance database. It could be that these income offenses are specific to the social deviance and disinhibition category of psychopathy—a culmination of affective, interpersonal, and behavioural deficits that include antisocial behaviours, aggression and several social-emotional difficulties [39]. While the similarities in these types of crimes may explain why they were the few that demonstrated relations to baseline ER, it should be noted that mean differences in ER were very small, calling into question the clinical significance of statistically significant findings in a large sample. Further, it is unclear why these relations emerged only at the 12-month follow-up, with no such links between ER and any type of crime observed at other timepoints. The current study sought to conduct exploratory, rather than confirmatory, hypotheses regarding the relation between ER and type of crime; future research should seek to replicate and expand upon these findings given that the presence of such an effect at only the 12-month timepoint (alongside the other concerns aforementioned) require cautious interpretation.

Several limitations to the present study should be noted. First, the study relies on the self-reported offending rates provided by juvenile offenders. Thus, scores could have been subject to fabrication, dismissal, and disengagement. Further, the distribution of self-reported offending necessitated dichotomizing in order to conduct hypothesis testing—data was dichotomized due to problematic skewness and kurtosis for all SRO continuous variables. A great deal of information is lost when data is dichotomized; in the present study, individual recidivism rates for each timepoint were lost after dichotomizing SRO. The range of variation can be underestimated as a result of dichotomization of data; for example, participants that reoffend only one time are categorized in the same group as participants who reoffend twenty times. Additionally, dichotomizing data can hide any non-linearity between variables and outcomes [40]. The inability to use a continuous version of SRO has the possibility of depressing the dependent variable, which may have been the reason for our null findings; mean differences can be seen between continuous SRO and dichotomous SRO in Table 1.

The 88 *t*-tests conducted for types of crime may have been subject to type 1 error and could be a source of inflation. Additionally, the current study utilized ER scores only from baseline interviews and did not use ER scores from each timepoint because the aim was to examine the predictive ability of ER over time. However, ER abilities are known to change across adolescent development [41] and thus, our reliance on baseline ER may have obscured important developmental changes in ER that may be predictive of recidivism. It may be of benefit to utilize ER scores obtained at the same timepoint as SRO for future analyses. As noted above, another possible limitation was the sample of adolescents utilized in this study. All adolescents had committed a criminal offense prior to participating in this study—group means may have been too similar given low ER (compared to the general population) among all juvenile offenders, regardless of reoffending. Additionally, the seven-year timeframe may have affected the results as adolescence is a pivotal phase for growth and development. Lastly, the current study aimed to predict a relatively low base rate event (i.e., criminal offenses), which has been shown to be difficult to do because it introduces small-sample bias [41].

The present study also had several strengths. First, our study included a diverse sample of adolescent offenders (*n* = 1354) who were each followed for a seven-year period—utilizing data from a large longitudinal study. Second, this is one of the only studies to consider ER and crime broadly in a population of serious juvenile offenders, rather than focusing on specific emotions within the ER category (i.e., anger or jealousy). Emotions like anger are important to consider with regards to ER and crime; however, other emotions (i.e., jealousy, irritability or depression) may play an important role as well and should be considered in future research [3]. Finally, this study sets a basis for future research studies on ER and juvenile delinquency.

## 6. Conclusions

In sum, the present study aimed to examine a hypothesized relation between ER and juvenile delinquency with the broader goal of enhancing current crime prevention efforts and clinical treatments for juvenile offenders—by identifying how the individual factor of DER may have played a role. Study findings failed to provide evidence that ER was a significant predictor of recidivism (dichotomized) over time but did suggest that ER is related to participation in certain types of crime one year later. Future directions should include the use of reliable and psychometrically sound measures for ER—utilizing a questionnaire that measures the ability to identify, understand and process emotions, rather than solely the ability to change an emotional experience—in future efforts to examine links between ER and recidivism. Additionally, there was a clear difference in sample characteristics between the present study and previous studies. Future studies may benefit from utilizing a two-group sample, where one group will represent juvenile offenders, and the other will represent juveniles that have not previously offended. Lastly, all timepoints and scores of ER should be utilized with their corresponding timepoints and scores of crime rate (SRO); for example, ER at baseline with SRO at baseline, then ER at 6-months with SRO at 6 months, etc., given that ER likely reflects a time-varying covariate of reoffending. These directions for future research should be undertaken in service of the broad aim of expanding preventative and intervention efforts for justice involved youth. The findings from examining this predictor have the potential to enhance current crime prevention efforts and clinical treatments for juvenile offenders by identifying how the individual factor of dysregulated ER may play a role. If it is a predictor, it would allow health care providers to identify children who may suffer from maladaptive ER and provide them with the proper care.

## Figures and Tables

**Table 1:** Descriptive Statistics.

Variables		Mean/Percent	SD	Min.	Max.	*N*
Age of first onset		10.42	1.81	9	17	1343
Sex	Male	86.4	-	-	-	1170
	Female	13.6	-	-		184
ER		2.76	0.659	1.00	4.00	1354
Self-Reported Offending (SRO)	6-months	34.77	194.50	0	3250	1260
	12-months	35.92	163.760	0	2062	1260
	18-months	47.89	215.90	0	2282	1228
	24-months	56.58	265.76	0	3986	1230
Dichotomous- SRO	6-months					
	Offended	54.2	-	-	-	734
	Did Not Offend	38.8	-	-	-	526
	12-months					
	Offended	47.6	-	-	-	644
	Did Not Offended	45.5	-	-	-	616
	18-months					
	Offended	42.8	-	-	-	579
	Did Not Offend	47.9	-	-	-	649
	24-months					
	Offended	40.5	-	-	-	548
	Did Not Offended	50.4	-	-		682

**Table 2:** Binary Logistic Regression Analyses Summary.

Variables	B	SE B	*β*	*p*	*X* ^2^
DV: 6 Month SRO				0.38	1.937
Gender	0.232	0.167	1.261	0.165	
ER	0.006	0.087	1.006	0.946	
DV: 12 Month SRO				0.124	4.179
Gender	−0.339	0.169	0.713	0.045	
ER	−0.02	0.086	0.98	0.813	
DV: 18 Month SRO				0.294	2.446
Gender	−0.042	0.168	0.959	0.501	
ER	0.134	0.087	1.144	0.121	
DV: 24 Month SRO				0.545	1.215
Gender	−0.069	0.166	0.933	0.678	
ER	−0.087	0.087	0.916	0.315	

**Table 3:** Independent-Sample *T*-test Summary.

	Mean ER Group 1	SD Group 1	Mean ER Group 2	SD Group 2	*t*	*p*	*d*
*12 Months*							
Destruction of property	2.76	0.66	2.79	0.66	−0.632	0.527	0.05
Set fire	2.76	0.66	2.67	0.86	0.394	0.69	0.12
Enter building to steal	2.76	0.66	2.77	0.62	−0.08	0.94	0.02
Shop lifted	2.76	0.66	2.75	0.64	0.13	0.895	0.02
Bought/received/sold stolen property	2.75	0.66	2.84	0.62	−1.74	0.08	0.14
Used check/credit card illegally	2.76	0.66	3.08	0.58	−2.01	0.045	0.52
Stole vehicle	2.75	0.66	2.95	0.6	−1.89	0.059	0.32
Sold Marijuana	2.75	0.66	2.81	0.62	−0.89	0.374	0.09
Sold other drugs	2.75	0.66	2.84	0.61	−1.39	0.164	0.14
Carjack	2.76	0.66	3.17	0.65	−2.16	0.031	0.63
Drove under influence	2.75	0.67	2.83	0.61	−1.51	0.132	0.13
Paid for sexual relations	2.76	0.66	3.37	0.51	−2.27	0.023	1.03
Shot someone	2.76	0.66	2.95	0.6	−1.25	0.211	0.28
Shot at someone (bullet hit)	2.75	0.66	2.94	0.64	−1.993	0.046	0.30
Robbed with weapon	2.75	0.66	2.93	0.63	−1.93	0.054	0.28
Robbed without weapon	2.76	0.66	2.79	0.6	−0.54	0.59	0.05
Beat someone up badly	2.76	0.66	2.76	0.66	0.042	0.966	0.00
Been in a fight	2.75	0.66	2.78	0.66	−0.858	0.39	0.05
Fight as part of gang	2.75	0.66	2.85	0.62	−1.33	0.184	0.16
Broken into car to steal	2.75	0.66	2.87	0.59	−1.37	0.17	0.19
Stole car to joyride	2.76	0.66	2.83	0.58	−1.05	0.292	0.11

*Notes:* Group 1 = did not offend, Group 2 = did offend.

## Data Availability

This study used publicly available data from the Pathways to Desistence study (“Pathways to Desistance”, 2000), a longitudinal and collaborative study that followed 1354 adolescent offenders for 7 years. The datasets generated and analysed during the current study are available in the pathways to desistance repository, https://www.pathwaysstudy.pitt.edu.
